# Metatranscriptome Analysis of Fig Flowers Provides Insights into Potential Mechanisms for Mutualism Stability and Gall Induction

**DOI:** 10.1371/journal.pone.0130745

**Published:** 2015-06-19

**Authors:** Ellen O. Martinson, Jeremiah D. Hackett, Carlos A. Machado, A. Elizabeth Arnold

**Affiliations:** 1 Department of Ecology and Evolutionary Biology, The University of Arizona, Tucson, Arizona, United States of America; 2 Department of Biology, University of Maryland, College Park, Maryland, United States of America; 3 School of Plant Sciences, The University of Arizona, Tucson, Arizona, United States of America; Institut National de la Recherche Agronomique (INRA), FRANCE

## Abstract

A striking property of the mutualism between figs and their pollinating wasps is that wasps consistently oviposit in the inner flowers of the fig syconium, which develop into galls that house developing larvae. Wasps typically do not use the outer ring of flowers, which develop into seeds. To better understand differences between gall and seed flowers, we used a metatranscriptomic approach to analyze eukaryotic gene expression within fig flowers at the time of oviposition choice and early gall development. Consistent with the unbeatable seed hypothesis, we found significant differences in gene expression between gall- and seed flowers in receptive syconia prior to oviposition. In particular, transcripts assigned to flavonoids and carbohydrate metabolism were significantly up-regulated in gall flowers relative to seed flowers. In response to oviposition, gall flowers significantly up-regulated the expression of chalcone synthase, which previously has been connected to gall formation in other plants. We propose several genes encoding proteins with signal peptides or associations with venom of other Hymenoptera as candidate genes for gall initiation or growth. This study simultaneously evaluates the gene expression profile of both mutualistic partners in a plant-insect mutualism and provides insight into a possible stability mechanism in the ancient fig-fig wasp association.

## Introduction

Mutualisms are ubiquitous in nature and often enhance the nutrition, protection, and/or reproduction of participating partners (see [1, 2]). Yet, mechanisms that maintain mutualisms largely remain unknown [3–5], in part because both partners typically incur a cost when providing a benefit to one another [6–8]. These costs create an inherent tension because selection shapes each lineage to maximize its own fitness by minimizing costs [9, 10]. In mutualisms characterized by vertical transmission (e.g., obligate bacterial endosymbionts and their insect hosts) these tensions are reduced because reproductive success of the symbiont is directly tied to that of its host [5]. However, in mutualisms with horizontal transmission (e.g., pollinator and plant mutualisms), the partners are often not as closely aligned, which can allow the exchange of costs and benefits to shift toward parasitism and a breakdown of the mutualistic association [11].

The obligate mutualism between figs (*Ficus* spp.) and fig wasps (Chalcidoidea, Agaonidae) arose >80 million years ago [12–15] and has long been of interest for addressing the mechanistic aspects and evolution of mutualistic interactions (reviewed in [16]; see also [17–19]). Figs are exclusively pollinated by fig wasps, which can only reproduce in fig flowers. Within the enclosed inflorescences of figs (i.e., syconia), a flower that receives pollen and a fig wasp egg will develop into a gall that will house a developing larva. Alternatively, a flower that receives only pollen will give rise to a fig seed. Because a single flower cannot yield both a fig wasp and a fig seed [20], an inherent tension exists: the fig requires both seeds and wasps to carry pollen to the next fig, but the ovipositing fig wasp (i.e., the foundress) does not gain an individual advantage by leaving flowers for seed development [14]. Indeed, reproductive success of the foundress could be greatest if it exploited all available flowers [20].

Yet, researchers have long noted that wasps consistently oviposit in only about half of the flowers available in a fig syconium (e.g., [17, 20]). This has led some authors to suggest that stabilizing mechanisms are used by the fig tree to control the fig wasp-to-seed ratio [19, 21, 22]. Such stabilizing mechanisms could explain the observation that regardless of the number of foundresses entering the fig, the number of emerging offspring remains relatively constant across syconia of the same species [17].

**Fig 1:**
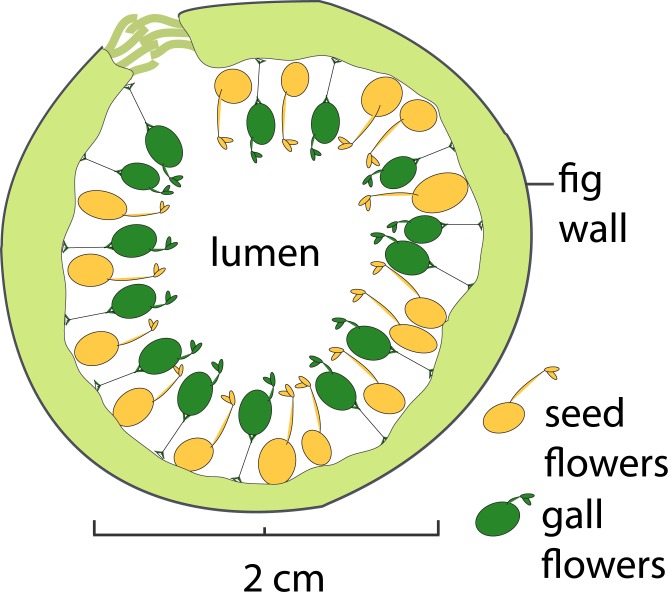
Schematic diagram of a cross section of a receptive syconium of *Ficus obtusifolia*. All parts of the syconium are drawn to scale, but flower density was decreased for this illustration.

Use of only a subset of available flowers by foundresses cannot be explained by physical limitations in ovipositor length or egg number [3, 5, 17, 23, 24]. It also does not reflect overt differences in the structure of the flowers: those receiving pollen and eggs (gall flowers) and those receiving only pollen (seed flowers) are structurally similar with regard to ovule shape and ovipositor access, and differ structurally only in terms of style length and stigma shape (Fig 1) [25]. Yet despite their structural similarity, flowers within a given syconium are not equally attractive to fig wasps [21, 25]: flowers located closer to the lumen of the syconium are strongly preferred for oviposition, whereas those near the fig wall are preferentially only pollinated [3, 22] (Fig 1). These flowers are referred to as ‘gall flowers’ and ‘seed flowers,’ respectively, based on their position, use by foundresses, and the ultimate production of galls or seeds in mature syconia.

Based on observations that foundresses are selective in choosing flowers for oviposition, West and Herre [21] proposed the ‘unbeatable seed’ hypothesis, suggesting that seed flowers may be physiologically incapable of developing into galls or do not support larval development after gall induction [21]. Evaluating this hypothesis, and understanding the dynamics of fig-fig wasp interactions more generally, requires an understanding of factors 1) motivating oviposition choice by foundresses, and 2) influencing gall induction. However, both the unbeatable seed hypothesis [24] and potential mechanisms underlying it have yet to be tested directly. In particular, whether subtle differences in gall- and seed flowers drive oviposition choice remains unknown, and potential mechanisms by which foundresses induce gall formation have not been identified.

The aim of this study is to examine potential differences in gall and seed flowers in developing figs, with particular attention to differences in gene expression that can provide insight into oviposition choice and gall induction. To do so we sequenced mRNA of flowers located near the lumen wall (putative seed flowers) and in the interior of the lumen (putative gall flowers) in developing syconia of *Ficus obtusifolia*, a monoecious fig that grows natively in lowland forests in Panama. Specifically we compared metatranscriptomes of gall and seed flowers before and after fig wasps entered syconia to determine (1) biochemical and metabolic differences between these two flower types that may influence oviposition by foundress wasps, and (2) gene expression in floral tissue at gall initiation [26]. This metatranscriptome analysis examines expression by all eukaryotic organisms present within flower tissue, and represents the first metatranscriptome of fig-fig wasp interactions. By providing a first perspective on gene expression relevant to oviposition choice and galling, which are essential to the fig and fig wasp interaction, we aim to develop hypotheses regarding the stability of this ancient and remarkable mutualism.

## Materials and Methods

This study was conducted at Barro Colorado Nature Monument, Panama (BCNM; 9°9’N, 79°51’W; 25m above sea level; for a full site description see [27]). A terrestrial and marine collection and export permit was obtained from the National Environmental Authority (ANAM) of Panama for the collections in this study.

Flowers of wild *Ficus obtusifolia* (subgenus *Urostigma*, section *Americana*) were collected in January-February 2010. Dozens of trees were surveyed over several months to identify two trees with compatible timing, defined as one releasing pollinating wasps (i.e., a source tree) and the other having nearly receptive syconia (i.e., a recipient tree) at the same time. Selected trees were mature individuals located ca. 8.6 km apart, which is within the natural flight range of foundresses [28].

Syconia of the recipient tree were covered individually with fine mesh bags to exclude pollinating and non-pollinating fig wasps. When syconia on the recipient tree became receptive, a single foundress (i.e., one *Pegoscapus hoffmeyerii* female), obtained from the source tree, was introduced to each fig. Each recipient syconium was then re-bagged for 24 hours to ensure pollination and galling by only a single individual with a known entrance time.

### RNA isolation and sequencing

**Fig 2:**
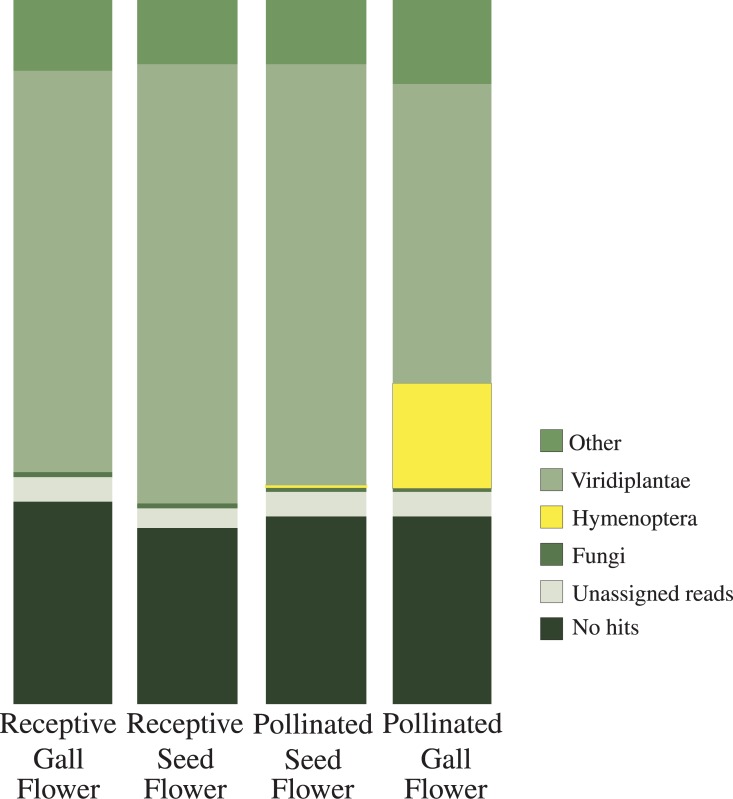
Taxonomic placement of assembled transcripts for each flower type. Columns indicate the percent of total transcripts assigned by MEGAN to Viridiplantae, Hymenoptera, fungi, and other organisms, as well as transcripts that had no annotation.

A ‘receptive’ sample of fig syconia was collected from the recipient tree prior to the introduction of foundresses, and a ‘pollinated’ sample of fig syconia was collected 24 hours after foundress introduction, when the first signs of gall growth could be observed. Syconia were submerged immediately in RNAlater (Qiagen) and held at -20°C until flowers were collected. Putative gall and seed flowers were identified only by their location (nearest to the lumen or nearest to the syconium wall, respectively) (Fig 1), and not by style length or stigma shape. This selection process was validated by the absence of sequenced wasp mRNA in pollinated seed flowers (Fig 2).

Flowers were removed with sterile microforceps, pooled with hundreds of flowers of the same position and pollination status from 2–3 additional syconia from the same recipient tree, and stored separately in RNAlater at -80°C to yield four types of samples: 1) receptive gall flowers, 2) receptive seed flowers, 3) pollinated gall flowers, and 4) pollinated seed flowers.

Before extraction, each sample was soaked in RNase-free water for 1 minute to remove residual RNAlater and then ground in liquid nitrogen. Total RNA was extracted from each of the four sample types using the Spectrum Plant Total RNA Kit following manufacturer's instructions (Sigma-Aldrich, St. Louis, MO) and visualized on an Agilent 2100 Bioanalyzer (Santa Clara, CA) to assess quality. cDNA library creation and sequencing was performed at the University of Arizona Genetics Core (UAGC) using the Illumina RNA-Seq TruSeq protocol on a HiSeq 2000 (San Diego, CA) with paired 100 bp reads with a 300 bp average insert length. The four resulting libraries were pooled and run on two-thirds of one lane.

### Assembly and mapping

After assessing read quality in FastQC version 0.10.0 (www.bioinformatics.bbsrc.ac.uk/projects/fastqc), we removed the first 10 base pairs of every read because they contained a skewed GC content relative to the rest of the read. Further trimming was performed using Trimmomatic version 0.22 (http://www.usadellab.org/cms/index.php?page=trimmomatic) where low quality reads (phred scores <15) were identified from both ends and across the entire read by a 15 bp sliding window. Reads shorter than 75 bp were removed. Raw reads from all samples are submitted to SRA accession SRP029217.

*De novo* assembly for each sample was performed using Oases version 0.2.08 [29]. Because all samples were collected from the same individual tree, all four samples also were combined and assembled to form a non-redundant reference metatranscriptome. We used the Oases “conserve long” option to assemble our final build using a kmer length of 51, and included five additional builds (kmers ranging from 31–51) as long sequences with the minimum contig length set to twice the kmer length [29]. Oases clusters highly similar sequences into groups likely derived from the same loci. The longest read in the group was used for subsequent analyses to prevent redundancy in mapping to the reference metatranscriptome.

Sequence reads from each sample were mapped to the reference metatranscriptome to quantify the abundance of each assembled transcript using the default parameters in CLC Genomics Workbench software (CLC bio, Denmark, last accessed November 10, 2012). The coverage of each transcript was determined in terms of the number of reads per kilobase per million (RPKM) reads mapped. The full data set expression values are deposited at GEO accession GSE55700 (http://www.ncbi.nlm.nih.gov).

### Annotation and taxonomic placement

For functional annotation of each transcript, all sequences were searched against the non-redundant protein database (nr) using BLASTx with a cutoff e-value < 1 x 10^−5^. Gene ontology (GO) terms were then assigned by BLAST2GO v2.5.0 [30]. GO terms were subsequently assigned to metabolic pathways according to KEGG mapping [31, 32].

Taxonomic assignments of transcripts were predicted using MEGAN v4.62.7 [33]. Each taxonomic assignment was performed using the lowest common ancestor (LCA) algorithm, which assigns each transcript to the lowest common ancestor in the NCBI taxonomy from a subset of the best scoring matches from BLAST. Although transcripts from plants were dominant in the data set, a diverse community of organisms was identified within these syconia, including wasps, fungi, and bacteria (Table A in S1 File).

Transcripts assigned to Hymenoptera were tested for the presence of signal peptides using the SignalP v4.1 server [34] and compared to the venom genes of *Nasonia vitripennis* using BLASTp with a cutoff e-value < 1 x 10^−5^ [35, 36]. *Nasonia vitripennis* was used because it is the only member of the superfamily Chalcidoidea to have a known venom composition.

### Statistical analyses

Due to difficulties in obtaining high-quality RNA in the field, we were unable to replicate our sample types for analysis. Therefore, we focus our analysis on transcripts that are significantly differentially expressed, and present in overrepresented gene ontology (GO) categories, with the aim of providing a first estimate of differences in expression between gall- and seed flowers before and after pollination. Each sample in this study is a pool of hundreds of individual flowers from multiple syconia, which averages biological variation among flowers in each category on the recipient tree. A previous study has shown that RPKM < 5 are unreliable in single replicate studies; therefore we only report transcripts with an RPKM value > 20 in at least one sample, which have been shown to be more robust [37].

Transcript abundance levels were analyzed with the CLC Genomics Workbench software to assess significant differences in expression levels based on RPKM. Expression levels were compared using the Kal test (a Z test) [38] with a p-value corrected for false discovery rate (FDR) to contrast gene expression by (1) receptive gall flowers vs. receptive seed flowers, and (2) pollinated gall flowers compared separately to pollinated seed flowers and receptive gall flowers. Overrepresented GO categories were determined using BiNGO v2.44 in Cytoscape version 2.8.2 [39]. Subsets of GO terms were compared to all the GO terms identified in the reference metatranscriptome.

## Results and Discussion

After trimming low-quality base pairs and reads, ca. 204.7 million clean paired-end reads remained for assembly. Samples averaged 51.2 million reads (Table 1). *De novo* assembly of clean reads resulted in an average of 36,092 (SE = 3,179) transcripts per sample. Pollinated gall flowers had the greatest number of assembled transcripts as they contained eggs of pollinating fig wasps in addition to plant tissue (Table 1, Fig 2).

**Table 1:** Sequencing and assembly statistics for each flower type.

	Total transcripts	Annotated transcripts	Total reads	Mapped reads (%)
**Receptive Seed**	32394	24091	17220340	67.3
**Receptive Gall**	32711	22950	72519250	70.8
**Pollinated Seed**	33669	24396	31455052	70.9
**Pollinated Gall**	45595	33031	83553656	70.6

Columns indicate number of assembled transcripts, transcripts annotated through BLASTx, and raw reads obtained from sequencing, as well as the percentage of raw reads that mapped onto assembled transcripts.

### Mapping and annotation

Cleaned reads were mapped for each sample to the reference transcriptome. Over half of the transcripts (51.2%, 27,929 transcripts) were present in all four samples (S1 Fig). An average of 2,612 reads covered each transcripts, but this was highly variable (SD = 6,772). A total of 30,166 of 54,550 transcripts (55.3%) had similarity to a known protein in the NCBI nr database, with a cutoff of e < 1 x 10^−5^. BLAST2GO assigned a functional annotation to 19,566 transcripts (35.9% of the total assembly). Taxonomy was assigned for 72.4% of the transcripts with an annotation (Fig 2).

### Distinguishing gall and seed flowers

Previous studies have shown that both pollinating and non-pollinating fig wasps strongly prefer to oviposit in the inner ring of flowers within the fig syconium (i.e., in gall flowers) [21, 22, 25] (Fig 1). Our results were consistent with these observations and indicated that in general we appropriately separated gall- and seed flowers on the basis of position alone. The vast majority of taxonomic assignments for transcripts from pollinated seed flowers were to Viridiplantae (85.9%), with few assignments to fungi (1.0%) and bacteria (2.6%). Around 13.0% were assigned to other taxa at < 1% each (e.g. alveolates and nematodes) or to internal nodes of the tree (Table A in S1 File). In contrast, in pollinated gall flowers nearly a quarter of the transcripts were assigned to Hymenoptera (21.3%) with the majority of the remaining assignments to Viridiplantae (60.8%) (Fig 2). A few transcripts in pollinated seed flowers (<0.1%) were assigned to Hymenoptera, which could be explained by a very few oviposition events in seed flowers or slight contamination from pollinated gall flowers during collection.

### Receptive gall flowers and seed flowers differ in expression

The preference by ovipositing wasps for the inner ring of flowers has been interpreted previously as evidence that fig trees limit foundresses to a subset of available flowers, allowing for development of both pollinators and fig seeds [21]. Under the unbeatable seed hypothesis this limitation is thought to reflect inherent structural or biochemical differences between gall flowers and seed flowers [21]. To determine whether flowers differ in a manner that could influence oviposition or wasp development, we compared gene expression of gall and seed flowers from syconia that were receptive to oviposition and pollination (i.e., before a foundress entered the syconium).

**Fig 3:**
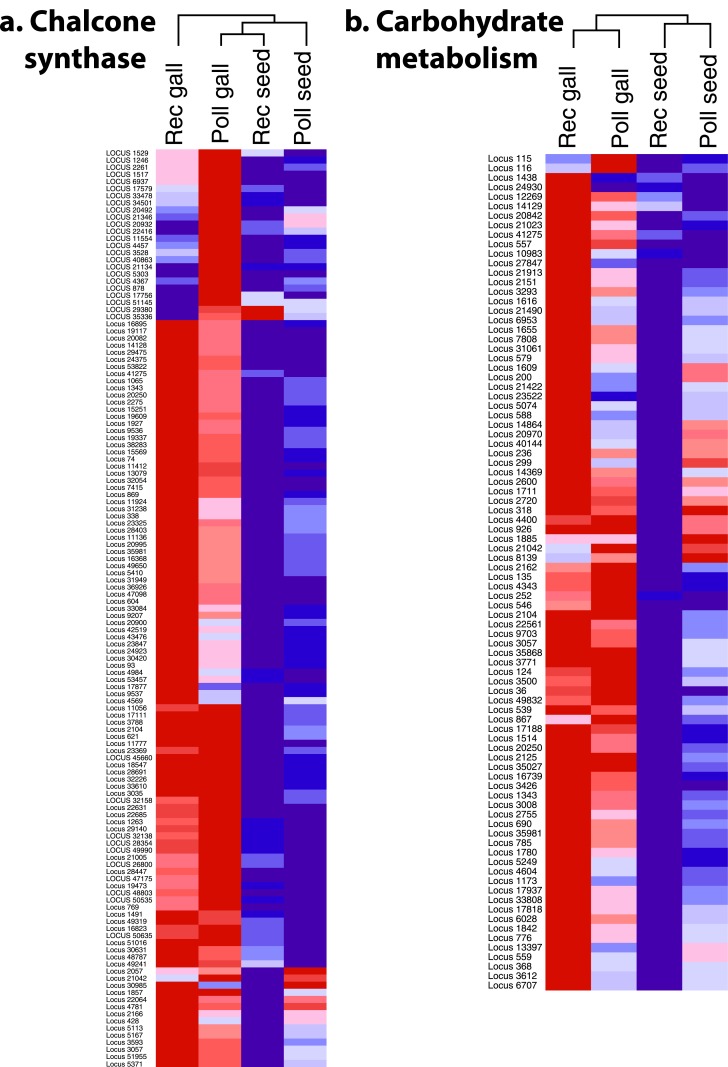
Clustered display of expression data from the metatranscriptome of *Ficus* flowers. Transcripts were selected for this analysis from overrepresented GO terms categories: a) Chalcone synthase and b) carbohydrate metabolism. The color scale ranges from saturated blue for log fold change -3.0 and below to saturated red for log fold change 3.0 and above the mean expression for each flower type. Each transcript is represented by a single row of colored boxes; each flower type is represented by a single column. Annotations for individual transcripts are available in Supplementary Tables 2–4.

We found that in receptive syconia, 4,707 transcripts (~10% of plant assigned transcripts) were significantly differentially expressed between the two flower types. This is the first evidence suggesting inherent differences between gall and seed flowers beyond position, style length, and stigma shape, in agreement with predictions from the unbeatable seed hypothesis [21]. Of these, 2,848 were up-regulated in gall flowers. Among these up-regulated transcripts, 127 GO terms were significantly overrepresented in the receptive gall flower compared to the reference metatranscriptome (Table B in S1 File). Transcripts assigned to L-phenylalanine metabolism, specifically chalcone synthase, were the most common, present in 11 overrepresented GO terms and making up 30% of the top 100 expressed transcripts in gall flowers (Fig 3a, Table C in S1 File).

All genes necessary to produce flavonoids from phenylalanine were significantly up-regulated in receptive gall flowers [40]. Flavonoids serve many functions in plants, including production of pigments and defense compounds and UV protection (see [41–43]). The roles that flavonoids play in plant-insect interactions also are diverse (reviewed in [44, 45]): they can be feeding stimulants or deterrents to insects [46–48], and stimulate oviposition in several species of Lepidoptera [49–51]. Functions of flavonoids within gall flowers cannot be determined here, but previous research has demonstrated that insects can detect flavonoids in plants and use this information for host selection [52]. Up-regulation of flavonoids in gall flowers could create a strong signal, detectable to the fig wasp, which could direct foundress wasps to oviposit in gall flowers. We propose this as a hypothesis for further study.

A large set of up-regulated transcripts in gall flowers, present in significantly overrepresented GO terms, were associated with sugar and carbohydrate metabolism (Fig 3b, Tables B and E in S1 File). On average, transcripts within these GO terms had a 5.1-fold increase of expression in gall flowers relative to seed flowers. Several transcripts assigned to sucrose, galactose and fructose metabolism (e.g., sucrose synthase, UDP-D-glucuronate carboxylyase, alpha-galactosidase and fructokinase-2) had high expression in gall flowers, suggesting gall flowers may have higher sugar content than seed flowers. In the domesticated fig (*Ficus carica*), concentrations of sucrose, galactose and fructose vary throughout fig development, peaking when figs are ripe; however, another peak occurs when the fig is receptive [53, 54]. Our data suggest the hypothesis that the higher nutritional quality of tissue in gall flowers may play a role in oviposition choice, with consequences for larval development. Seed flowers may lack the nutritional quality to support the development of a wasp larva, or may contain diminished nutritional resources with negative effects on larval development (Fig 3b). Consistent with this hypothesis, wasps that develop in seed flowers tend to be significantly smaller, less likely to be mated by male wasps, and have lower survival [3, 22, 55]. By providing a nutrient-rich subset of flowers for wasp development, figs could maintain female fitness by producing seeds from less nutritionally expensive seed flowers, and enhance male fitness by fostering more robust wasps that can carry more pollen over greater distances.

### Distinctive features of seed flowers in receptive syconia

Of the 4,707 transcripts that were differentially expressed between gall and seed flowers in receptive syconia, 1859 were up-regulated in seed flowers. In the top 100 expressed transcripts of seed flowers, 73% either had no putative annotation or were assigned an annotation of unknown/hypothetical protein (compared to 40% in gall flowers). Interpretations are limited by a lack of sufficient gene annotation, but we cannot fully rule out the possibility that the fig is actively defending seed flowers or making them less attractive to foundresses for oviposition.

### Response of gall flowers to oviposition

Expression profiles from pollinated gall flowers were compared to pollinated seed flowers because both flowers typically receive pollen from the foundress [56], but differ in that seed flowers are not subject to oviposition. Pollinated gall flowers also were compared to receptive gall flowers because gall flowers were shown to have different expression profiles than seed flowers. Only transcripts that were differentially expressed (FDR < 0.05) in both comparisons, thereby having unique expression in pollinated gall flowers, were used in further analyses.

A total of 910 transcripts were differentially expressed in pollinated gall flowers relative to both pollinated seed flowers and receptive gall flowers, and were identified as being potentially involved with the interaction between the fig flower and the wasp embryo or maternal secretion. The majority of these differentially expressed transcripts were up-regulated in pollinated gall flowers (55.8%; 508/910). Only 23% of the transcripts were down-regulated (209/910), and the remainder had mixed expression (i.e. up-regulated compared to pollinated seed flowers, but down-regulated compared to receptive gall flowers).

Among the up-regulated transcripts of pollinated gall flowers, 44 GO terms were significantly overrepresented (Table F in S1 File). Similar to receptive gall flowers, transcripts assigned to genes involved in flavonoid synthesis were the most common (present in 15 overrepresented GO terms; 34%) (Fig 3a). Chalcone synthase and isomerase also made up 20% of the top 100 expressed transcripts in pollinated galls. However, the up-regulated flavonoid pathway gene set in receptive and pollinated gall flowers does not completely overlap (Fig 3a). For example, the first gene in the flavonoid pathway, phenylalanine ammonia-lyase, had higher expression in receptive gall flowers, whereas some genes involved much later in the pathway (e.g. anthocyanin 5-aromatic acyltransferase) had higher expression in pollinated gall flowers.

High levels of flavonoids (specifically chalcone synthase) are present at the beginning stages of gall formation by diverse organisms, including *Agrobacterium tumefaciens*, *Plasmodiophora brassicae*, and root-knot nematodes, and are also observed in the nodules formed by *Rhizobia* [57–60]. During gall formation, flavonoids act as a transport inhibitor to auxin, a plant growth hormone, which allows its concentration to build in the galled tissue [57–60]. To the best of our knowledge, this is the first study to demonstrate a high expression level of transcripts assigned to chalcone synthase in an insect-induced gall.

Several transcripts were assigned to defense-induction genes in three over-represented GO terms (i.e. beta-galactosidase, chitinase, peroxidases, and beta-1,3-glucanase; their roles in plant defense are reviewed in [61, 62]). Syconia often harbor many organisms in addition to fig wasps (including bacteria, fungi, nematodes [63, 64]). This non-specific defensive response could play a role in protecting developing galls from pathogens or other antagonists. In turn, fig wasps, which have been co-evolving with fig trees for over 80 million years, are likely to have developed a tolerance for such non-specific defense—an hypothesis to be addressed in future work.

### Hymenopteran gene expression in pollinated gall flowers

Overall, 7,045 transcripts from pollinated gall flowers were assigned to homologs from Hymenoptera (average coverage, 10.5x) (Table G in S1 File). Central metabolic pathways are relatively complete, such as the oxidative phosphorylation pathway (80.0% complete with 68/85 genes) or the citrate cycle pathway (91.3% complete with 21/23 genes). The number of Hymenopteran genes found was less than the 11,412 protein-coding genes in the genome of *Ceratosolen solmsi* [65], another fig-pollinating wasp; this is not surprising given that our data only include expression during the first 24 hours of wasp development.

The hymenopteran transcript with the highest level of expression was assigned to the gene *Giant* (*gt*) (RPKM = 9238.3, and ~3500 reads more than the next highest transcript). *Giant* is a maternally expressed gene that represses expression of the trunk gap genes so that the head and thorax form properly [66]. Additional transcripts with annotations to embryonic development were present among the highest expressed transcripts (e.g. *lethal(2)essential for life* **[** *l(2)efl*]). The high level of expression of these genes suggests that fig wasps were undergoing embryogenesis when gall flowers were collected.

A maternal secretion, which is deposited in every flower that receives an egg, has been associated with the initiation of gall growth in fig flowers [25, 67, 68]. However, the mechanism by which this secretion initiates gall growth is unknown. Growth continues in the presence of wasp eggs, which may share some gene expression with the maternal secretion that is important for gall growth. Therefore, genes that 1) potentially can be secreted from the egg as proteins and 2) shared by the venom gland (where the maternal secretion is produced) are potential candidates for gall initiation and growth.

Only 196 (2.8%) of the Hymenopteran transcripts observed here contain a signal peptide (Table G in S1 File). More than a third (34.2%) are assigned to hypothetical or unknown proteins. The top-expressing, annotated signal peptide protein (79^th^ highest expressing in all Hymenoptera transcripts) was assigned to icarapin, which has an active role in honeybee venom [69]. The function of icarapin is unknown, however it does produce an icarapin-specific IgE-response in humans [69]. When compared to proteins produced by the venom gland of *Nasonia vitripennis*, the only well-studied venom among wasps in the superfamily of Chalcidoidea, eight genes coding for proteins harboring signal peptides also shared homology with genes coding for venom proteins. Three are hypothetical proteins and the remaining five match genes predicted as serine protease, lysosomal acid phosphatase, chymotrypsin, lipase, and metalloproteinase (Table G in S1 File). Metalloproteinase has previously been identified as an effector in saliva of aphids and venom of the chalcid wasp, *Eulophus pennicornis* [70–71]. Additionally, plant matrix metalloproteinases are involved in plant growth, morphogenesis, and development [71]. Association with venom glands in other species and the presence of signal peptides suggest that these genes may be important in the fig-fig wasp association with regard to gall formation and maintenance.

## Conclusions

This study is the first to simultaneously describe and quantify the gene expression profile of both mutualistic partners in a plant-insect mutualism. Although replication and further experimental data are necessary to confirm quantification, the work presented here provides insight into gene expression relevant to oviposition choice and gall initiation in the fig-fig wasp mutualism. These analyses have revealed for the first time detectable differences in metabolism between gall flowers and seed flowers before oviposition. Detection of gene expression relevant to galling and defenses provides a new perspective on the dynamics within syconia before and after pollination. Our results suggest that differences between nutrient-rich and nutrient-poor flowers may help explain the balance of the seed-to-wasp ratio, long discussed in the study of this classical mutualism—and provide a suite of ideas ripe for further exploration.

## Supporting Information

S1 Fig Venn diagram displaying the number of assembled transcripts shared between samples. (PDF)

S1 File Tables from the Ficus metatranscriptome analyses. Number of transcripts assigned to taxonomic groups for each sample (Table A). Overrepresented GO terms of up-regulated genes in receptive gall flowers compared to receptive seed flowers (Table B). Transcripts assigned to overrepresented GO terms associated with chalcone synthase in receptive and pollinated gall flowers (Table C). Transcripts uniquely expressed in receptive gall flowers (Table D). Transcripts assigned to overrepresented GO terms associated with carbohydrate metabolism in receptive gall flowers (Table E). Overrepresented GO terms of up-regulated genes in pollinated gall flowers compared to receptive gall flowers and pollinated seed flowers (Table F). Transcripts with assignment to Hymenoptera (Table G).
(XLSX)
