# Supplementary material for: Metatranscriptome Analysis of Fig Flowers Provides Insights into Potential Mechanisms for Mutualism Stability and Gall Induction
Source: PLoS One. 2015 Jun 19;10(6):e0130745. doi: 10.1371/journal.pone.0130745 (PMC4474661; doi:10.1371/journal.pone.0130745)

Supplemental Figure 1 – Venn diagram displaying the number of assembled transcripts shared between samples.

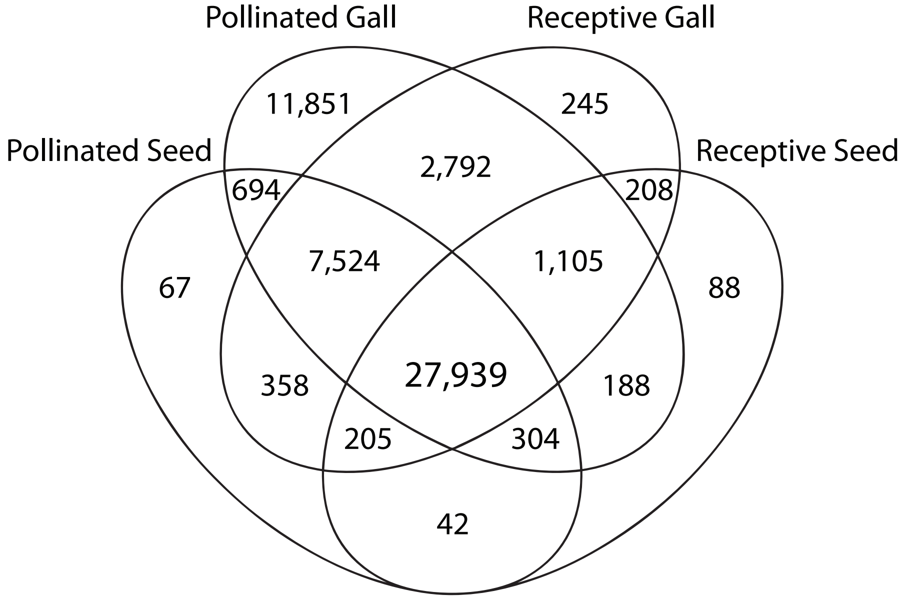

Supplement: S1 Fig — (PDF) [file pone.0130745.s001.pdf]
